# Reply to: Multiple induced seismicity mechanisms at Castor underground gas storage illustrate the need for thorough monitoring

**DOI:** 10.1038/s41467-022-30904-5

**Published:** 2022-06-17

**Authors:** Simone Cesca, Daniel Stich, Francesco Grigoli, Alessandro Vuan, José Ángel López-Comino, Peter Niemz, Estefanía Blanch, Torsten Dahm, William L. Ellsworth

**Affiliations:** 1 GFZ German Research Centre for Geosciences Potsdam, Potsdam, Germany; 2 Instituto Andaluz de Geofísica, Universidad de Granada, Granada, Spain; 3 Departamento de Física Teórica y del Cosmos, Universidad de Granada, Granada, Spain; 4 Department of Earth Sciences, University of Pisa, Pisa, Italy; 5 National Institute of Oceanography and Applied Geophysics - OGS, Trieste, Italy; 6 Institute of Geosciences, University of Potsdam, Potsdam-Golm, Germany; 7 Departament de Física-EPSEB, UPC Barcelona Tech, Barcelona, Spain; 8 Observatori de l’Ebre (OE), CSIC - Universitat Ramon Llull, Roquetes, Spain; 9 Department of Geophysics, Stanford University, Stanford, CA USA

**Keywords:** Seismology, Geophysics

**replying to** Vilarrasa et al. *Nature Communications* 10.1038/s41467-022-30903-6 (2022)

We welcome the call for seismic monitoring infrastructures around potential sources of anthropogenic seismicity^1^ in response to our report about seismicity at the Castor gas reservoir^2^. The lack of a proper seismic monitoring at the Castor site was previously recognized^3^ and potential monitoring solutions for offshore industrial operations proposed^3^. Our case of a successful analysis and interpretation despite poor instrumentation does not, of course, imply that a poor instrumentation is desirable. Independent of the setting and the analysis tools involved, more data and shorter recording distances generally allow for a better resolution of focal parameters and the identification of more details of seismicity. In particular, routine and near-real-time monitoring efforts depend heavily on the quality of the recording network to detect microseismic activity. On the other hand, we disagree with the technical comments about source depth and triggering mechanism^1^, suggesting that significant uncertainties may hinder the identification of the drivers of those seismogenic processes. We extensively quantified, reported, and discussed seismic parameters uncertainties^2^. We aimed to understand partially discrepant results of previous works, where uncertainties were rarely reported^4–8^. Out of our extended analysis of seismicity two sets of results are disputed^1^: the hypocentral depths and the mechanisms leading to seismicity. These issues are discussed below.

First, the comment^1^ states that large earthquakes nucleate at larger depths. We located the seismicity at 3–5 km depth, better constraining the more extensive range of previous estimates^4–7^, finally suggesting depths of ~3–4 km, to further account for relocation and centroid moment tensor inversion results. It may be typical for large earthquakes in the continental lithosphere to nucleate at medium depths within the earth’s crust^9^. However, there are notable exceptions here too, such as the 1992 Mw 7.3 Landers, nucleating at 3–6 km^10^, or the 2020 Mw 6.5 Monte-Cristo Range earthquake, where the mainshock depth was 3.74 km below the mean station elevation (~2 km b.s.l.)^11^ to cite just two. However, our main objection is that magnitude 4 earthquakes like those at Castor can hardly be considered large earthquakes. Such events are classified as small or moderate in the seismological literature. At Castor they involved ~1 km^2^ of rupture^2^, with propagation that is little affected by the primary stress gradient within the crust. Seismic catalogs in areas of dense instrumentation show that magnitude 4 events at depths about 3 km are quite common^12–15^, corroborating that there are no objections from physics to the existence of shallow earthquakes of this size. The Californian seismic catalog for the years 1985–2021 (Northern and Southern California Earthquake Center), for example, includes more than 250 earthquakes of magnitude *M* ≥ 4 with a depth shallower than 4 km^12–14^. The 2017 ML 4.3 Château-d’Oex earthquake, among the largest occurring in Switzerland over the last years, had a depth of 4 km, well constrained by P and S onsets recorded at only 3 km distance^15^. The case of induced seismicity is even more striking, as this is favored at shallow depths, where stress and pore pressure conditions are more easily altered by shallow underground operations^3^. Examples of earthquakes induced by fluid injection at shallow depth include the Mw 5.6 2011 Prague, Oklahoma, earthquake (depth 4 km)^16^ and its aftershock sequence (mostly with depth <5 km and early aftershocks within the sedimentary layers)^17^, earthquakes of up to Mw 5.3 in the Raton Basin, Colorado (mostly with depths 1–4 km)^18^, the multiple Mw ≥ 4 Timpson, Texas earthquakes (depths 1.6–4.6 km)^19^ or the Mw 5.4–5.5 2017 Pohang, South Korea, earthquake (depth 4.2 km)^20^. The Raton Basin and Timpson earthquakes occurred exclusively in the sedimentary section, while the Prague earthquake faulted both sediments and the underlying basement.

Our depth estimates were based on the t_pP_-t_P_ differential time between seafloor-reflected pP phase and direct P arrival. Records at multiple seismic stations constrain such delay to 1.5–1.8 s for the largest earthquakes at Castor. Converting these delays into a source depth requires knowing the velocity structure above the source. This can introduce epistemic uncertainty additional to aleatory uncertainty associated with the measurement of t_pP_-t_P_. For this reason, we tested a broad range of 1D velocity models and openly reported the estimated depths^2^. The suggested^1^ depth assessment using two additional 1D velocity models^7^ confirms a shallow focus (2–4 km). Hypocentral relocation and waveform similarity^2^ further constrain the seismicity within a narrow depth range. If depth would have been as large as 6–10 km, explaining differential t_pP_-t_P_ times^2^ of ~1.65 s would require an unrealistic average P wave velocity of 7.3–12.1 km/s.

Besides the depths, also epicentral locations have been a matter of debate in the past^2^. A new location comparison is here illustrative (Fig. 1): our relocations^2^ (Fig. 1b) improve the resolution of the NE-SW fault geometry, compared to absolute locations^7,21^ (Fig. 1a, c). Absolute locations can partially reconstruct the lateral distributions of the high waveform similarity clusters^2^ along the NE-SW direction but not along the NW-SE direction. This direction roughly corresponds to the orientation of the largest epicentral uncertainties (median orientation 122°)^5^, controlled by the asymmetric network geometry. Note that the epicentral locations (Fig. 1d) used by the comments’ authors to suggest a different fault geometry^8^ differ substantially from those they cite as source^5,7^, questioning their overall interpretation.

**Fig. 1:**
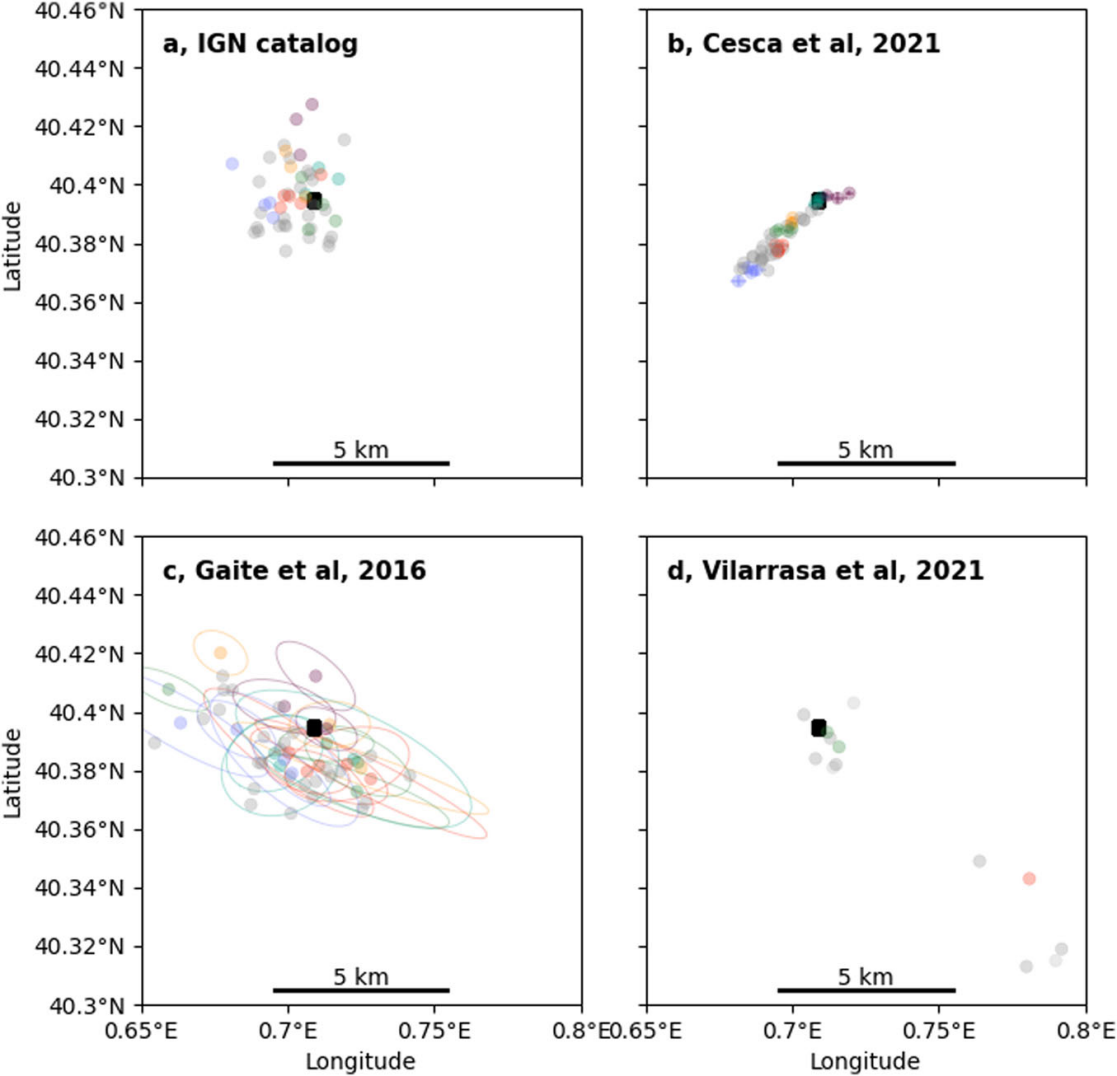
Comparison of epicentral location maps. **a** Catalog of the National Geographic Institute of Spain (IGN), **b** relocation based on cross-correlations^2^, **c** absolute locations using a 3D model^5^, and **d** subset of 13 events^8^ referred to such catalog^5^ but with discrepant locations. Epicenters are plotted for a common dataset of 49 earthquakes with magnitudes larger than 2, out of the 51 for which a waveform-based classification was performed^2^, except in (**d**), where we only plot the 13 available events in such dataset. Colored epicenters correspond to different families of earthquakes with high waveform similarity, which should be tightly located. Uncertainties are reported for the clustered events in (**b**) (as latitude and longitude bars) and (**c**) (ellipses). A black square marks the location of the Castor platform.

Regarding the mechanisms controlling the seismicity, we welcome the comments to be open to different plausible triggering mechanisms, including buoyancy, stress transfer, or poromechanical effects^1^. However, these mechanisms came into play to explain large source depths^5,7^ that would place the Castor seismic series within the crystalline basement underneath the reservoir. According to our relocations, the earthquakes occur within the sediments, in a location where a hydraulic connection to the reservoir is more plausible. The balance of evidence indicates that the characteristic speed-limited migration of seismicity follows a diffusion process. The migration was resolved using robust and well-recognized seismological techniques, namely hypocentral relocation^22^ and template matching^23,24^. The hydraulic diffusivity at Castor is poorly known. A value of 0.5 m^2^/s, which is not unusual^25–27^, was based on the observed migration of seismicity^2^. The comment’s claim that diffusivity should be substantially smaller is not supported by any reference^1^. Further, the comment states that ‘hydraulic connection between the storage formation and the depth of the earthquakes requires the existence of some unknown high permeability conduit or fault.’^1^. Conversely, several faults are reported close to the reservoir^6^ and some of them could have facilitated diffusion through permeable damage zones. Specifically, the Amposta fault bounds the reservoir and extends deeper^6^, next to the fault activated by the seismicity^2^. A hydraulic connection between the reservoir and greater depths is also suggested by pressure measurements made during the exploitation of the former Castor oil field^6^. The pressure decreased moderately between 1973 and 1976, during peak oil production, and then increased gradually accompanying the drop of the production rate, indicating aquifer support from below. Therefore, it is not surprising that raising the pressure in the reservoir during gas injection would communicate pressure below it. The largest earthquakes occurred with delays of ~20 days after an injection of 15 days. However, we attributed the seismicity to pore pressure diffusion and to asperities loading. The largest earthquakes did not occur when the pressure front reached their location, but were delayed as the later failure of unbroken, loaded asperities^2^. Other processes might have contributed to induce seismicity^1^. For example, the effects of buoyancy have been invoked^8^. However, it remains to be proven whether such a model can explain the NE-SW spatial distribution of seismicity at Castor and its migration: indeed, the disputed distribution of seismicity and the uncertainties in location, depth, and focal mechanisms were ignored when assessing that model^8^.

## Data Availability

Seismic data (catalogs) used in this study are available as Supplementary Dataset of our previous manuscript^2^, at the website of the Instituto Geográfico Nacional (IGN)^21^, or in the reference publication^5^, respectively. A fourth catalog^5^ may be available in full form upon request to the corresponding author, and a subset for 14 events is openly available^7^.

## Code availability

Reported analyses were performed in the cited publications.
